# The activity of Meniran (*Phyllanthus niruri* Linn.) extract on *Salmonella pullorum* infected broilers

**DOI:** 10.14202/vetworld.2022.1373-1382

**Published:** 2022-05-31

**Authors:** Sri Hidanah, Emy Koestanti Sabdoningrum, Kadek Rachmawati, Soeharsono Soeharsono, Gede Govinda Ananta Trika, Masy’ Ariel Huda, Tsania Putri Widiati

**Affiliations:** 1 Animal Husbandry Division, Faculty of Veterinary Medicine, Airlangga University, 60115, Surabaya, Indonesia; 2 Basic Veterinary Medicine Division, Faculty of Veterinary Medicine, Airlangga University, 60115, Surabaya, Indonesia; 3 Veterinary Anatomy Division, Faculty of Veterinary Medicine, Airlangga University, 60115, Surabaya, Indonesia; 4 Department of Basic Veterinary Medicine, Faculty of Veterinary Medicine, Airlangga University, 60115, Surabaya, Indonesia

**Keywords:** broiler chicken performance, food security, *Phyllanthus niruri* Linn, *Salmonella pullorum*

## Abstract

**Background and Aim:**

Pullorum is an acute and chronic disease caused by *Salmonella pullorum*, often infecting chicken farms. Pullorum disease treatment using antibiotics that do not follow the control dose can cause bacteria to become antibiotic-resistant. Meniran contributes to inhibiting and antagonizing bacteria and can increase the efficiency of chicken feed because of its bioactive compounds, including alkaloids, flavonoids, tannins, and saponins. This study aimed to determine the activity of Meniran extract (*Phyllanthus niruri* Linn.) in broilers infected with *S. pullorum*.

**Materials and Methods:**

*In vitro* study that was conducted includes phytochemical test, diffusion, and dilution methods using Meniran extract at 5%, 10%, 20%, and 40% concentrations and tylosin at 2% concentration. The data of the dilution method (minimum inhibitory concentration [MIC] and minimum bactericidal concentration [MBC]) were processed using probit analysis to determine LC_50_. *In vivo* study was conducted by randomly dividing 20 broilers into five treatment groups, four per group. The chickens (except in group P0−) were infected with *S. pullorum* aged 14 days. Then, the treatment was conducted according to the divided groups when the chickens were aged 21-34 days. The said treatments are P0− (uninfected *S. pullorum* and unadministered with Meniran extract), P0+ (infected with *S. pullorum* and unadministered with Meniran extract), and P1, P2, and P3 (infected with *S. pullorum* and administered with Meniran extract with 5%, 10%, and 20% concentrations, respectively). Data from the phytochemical test were analyzed as descriptive. The data from the diffusion method were analyzed using one-way analysis of variance (ANOVA) and Duncan’s test. Then, the results of broilers’ performance were analyzed using ANOVA and Duncan’s test.

**Results:**

The phytochemical test showed positive for alkaloid, tannin, saponin, flavonoid, and steroid/triterpenoid. The diffusion method formed the largest zone at 40% concentration with 15.6 mm, while 20%, 10%, and 5% had average of 13.15 mm, 8.38 mm, and 5.8 mm, respectively. The dilution method (MIC and MBC) exhibited the antibacterial ability of Meniran extract against *S. pullorum* at 20% dose and LC_50_ 14.118% concentration. The Meniran extract administration in broilers exhibited improved performance of chickens infected with *S. pullorum*, with the administration of 20% dose of Meniran extract showing the best result.

**Conclusion:**

About 20% concentration Meniran extract can serve as an antibacterial agent and showed the best results in broilers infected with *S. pullorum*.

## Introduction

Pullorum is a disease that often infects chicken farms. It is an acute and chronic disease caused by *Salmonella pullorum*. Pullorum disease in chickens shows several symptoms, such as closed eyes (sleepy eyes look alike), bluish combs, and decreased appetite. Usually, it exhibits white or greenish-brown diarrhea and the existence of lumps like paste around the cloaca accompanied by weakness of the legs, dangling wings, dullness, paralysis due to arthritis, and shortness of breath. Meanwhile, *S. pullorum* transmission can occur vertically and horizontally, between two or more cages [1-3].

Pullorum disease can be treated using antibiotics, including amoxicillin, enrofloxacin, and tylosin. However, any treatment exploitation using antibiotics that do not follow the control dose can cause bacteria to become antibiotic-resistant; thus, it will complicate the process of preventing and treating the disease [4,5].

Herbal medicine is an alternative used to prevent pathogenic bacterial infection. Meniran (*Phyllanthus niruri* Linn.) is a herbal plant often used for medicine. Meniran extract contains antibacterial substances; therefore, it can be used to inhibit bacterial growth. Besides, this extract also consists of bioactive compounds that have antibacterial activity from terpenoids, alkaloids, flavonoids, saponins, and tannins. Consequently, the provision of herbal plants can allegedly stabilize chickens’ health conditions and increase the efficiency of chicken feed [6-8].

Specifically, the Meniran herbal plant’s active substances inhibit and kill the bacteria, including alkaloids, flavonoids, tannins, and saponins. For example, alkaloids serve as antibacterials by damaging the peptidoglycan constituent components in bacterial cells. Accordingly, the bacterial wall layers will not be formed completely, leading to bacterial cell death. Meanwhile, flavonoids work by denaturing the proteins in the cell walls, consequently changing the structure and mechanism of permeability in the cell walls of bacteria. Furthermore, tannins serve to inactivate the enzymes and interfere with cell protein transport. As for saponins, its mechanism damages the bacteria’s cell membranes, releasing important components, such as proteins, nucleic acids, and nucleotides; thus, bacteria turn into lysed [9-13].

Previous studies have discussed the antibacterial activity of Meniran against various bacteria [14,15], but none has discussed its antibacterial activity against *S. pullorum*. This study aimed to determine the activity of Meniran extract (*P. niruri* Linn.) in broilers infected with *S. pullorum*.

## Materials and Methods

### Ethical approval

This study was approved by the Faculty of Veterinary Medicine, Airlangga University, Surabaya, Indonesia (Approval no: 346/UN3/2020).

### Study period and location

The study was conducted during May 2021 and June 2021 at several places in the Veterinary Medical Faculty of Airlangga University, such as the Animal Cage Unit, Bacteriology and Microbiology Laboratory of Microbiology Department, Molecular Biology Laboratory, and Pharmacology Laboratory of Basic Medical Science Department.

### Making of Meniran extract and Meniran extract concentration

Meniran (*P. niruri* Linn.) plants (10 kg) from Tawangmangu, Central Java, Indonesia, were identified by Fauzi from the Center for Research and Development of Medicinal Plants and Traditional Medicines, Research and Development Agency, Ministry of Health of the Republic of Indonesia (voucher specimen: B2P2TO-OT). The plants were dried in an open and shady place for 7 days [16]. Then, the dried Meniran was ground and sieved to obtain 1 kg Meniran powder form. Then, 1 kg Meniran powder was soaked for 3 days with a maceration procedure using 3 L 96% methanol solution and stirred once daily for 3 days [17]. Finally, the immersion of Meniran powder was squeezed using a flannel cloth, and the extract was evaporated using a rotary evaporator at a temperature of 500°C at 0.14 ×g speed.

This study implemented Meniran extract at doses of 5%, 10%, 20%, and 40%, in relation to the dosage of the extract [18]. The extract’s diluting process was performed from high to low concentrations. A 20% dose was made using 20 g concentrated Meniran extract, which was evaporated and dissolved in a diluent solution (0.5% Na CMC solution) until the solution reached 100 mL. Then, a total of 60 mL extract solution were put into a bottle and labeled with a 20% dosage, while the remaining 40 mL extract solution was dissolved again by adding 40 mL 0.5% CMC Na solution. Furthermore, the new solution was divided into two; 60 mL extract solution was put into a bottle and labeled with a 10% dose, while the remaining 20 mL extract solution was dissolved again by adding 20 mL 0.5% CMC Na solution. Finally, the final resulted solution of 40 mL was then put into a bottle and labeled with a 5% dose.

### Phytochemical test

#### Alkaloid test

The alkaloids test was conducted by implementing the method of Mayer, Wagner, and Dragendorff. Three milliliters of Meniran extract were placed in a porcelain cup, added with 5 mL 2 M HCl, then stirred and cooled at 25°C. Then, the cold sample was added with 0.5 g NaCl, then stirred and filtered. The filtrate obtained was mixed with three drops of 2 M HCl, then separated into four parts; A, B, C, and D. Filtrate A was used as a void, filtrate B was mixed with Mayer’s reagent (Merck, Germany), filtrate C was added with Wagner’s reagent (Merck), and filtrate D was used for the confirmation test. If a precipitate was formed on the Mayer and Wagner reagents added filtrates, it indicated the presence of alkaloids. In addition, a confirmation test was conducted by adding 25% ammonia into filtrate D to reach pH 8-9. Then, it was added with chloroform and evaporated over the water bath. The next step was adding 2 M HCl; it was then stirred and filtered. Then, the generated filtrate was divided into three parts; filtrate A was used as a void, filtrate B was tested with Mayer’s reagent, while filtrate C was tested with Dragendorff’s reagent (Merck). Any precipitate formation indicated the presence of alkaloids [19].

#### Tannin test with Denis’ reagent

Two grams of the Meniran extract were mixed into a 500 mL boiling flask, 350 mL of distilled water was added, and refluxed for 3 h. After cooling, the sample was transferred quantitatively into a 500 mL volumetric flask and then filtered. Two milliliters of the sample were collected and placed into a 100 mL volumetric flask. Folin Denis reagent (Merck) in 2 mL and 5 mL saturated Na_2_CO_3_ was added, then left for 40 min, and the absorbance was measured at a wavelength of 725 nm. Next, 100 g sodium tungstate (Merck), 20 g phosphomolybdic acid (Merck), and 50 mL 85% phosphoric acid (Merck, Indonesia) were mixed into 750 mL distilled water, then refluxed for 3 h, cooled, and added with distilled water to 1 L. Then, 3 g anhydrous Na_2_CO_3_ (Merck) was combined with 100 mL distilled water at 70-80°C temperature, stirred until it dissolved, then cooled off again overnight [19].

#### Saponin test

The saponin test was performed by applying the Forth method [19] by inserting 2 mL Meniran extract into a test tube, then added with 10 mL distilled water, shaken for 30 s, and changes were observed. If a solid foam was formed and did not vanish within 30 s, it indicated the presence of saponins. In addition, the saponin confirmation test was implemented by evaporating the sample to dry and washing it with hexane until the filtrate became clear. The remaining residue was added with chloroform, stirred for 5 min, then combined with anhydrous Na_2_SO4 (Merck) and filtered. The filtrate was divided into two parts, A and B. Filtrate A was used as a void, filtrate B was added with acetic anhydride, stirred slowly, mixed with concentrated H_2_SO_4_ (Merck)_,_ and stirred again. Finally, the formation of a red to brown ring indicated the existence of saponins [19].

#### Flavonoid test

The sample weighed 3 mL. It was evaporated and washed with hexane (Merck) until it became clear. Next, the residue was dissolved in 20 mL ethanol and filtered. The filtrate was split into three parts; A, B, and C. Filtrate A was used as a void, filtrate B was added with 0.5 mL concentrated HCl (Merck), then heated in a water bath; if any dark red to purple change occurred, it showed a positive result (Bate Smith-Metcalf method). Meanwhile, filtrate C was added with 0.5 mL HCl and Mg metal, then the color change was observed (Wilstatter method). Flavone compounds formed the existence of red to orange colors, while flavanols or flavanones constructed the dark red colors, and aglycones or glycosides triggered the green to blue colors [19].

#### Steroid and triterpenoid tests

Steroid and triterpenoid tests were performed by applying the Liebermann-Bouchard method. The reaction of triterpenoids with Liebermann’s reagent (Merck) produced a reddish-purple color, while steroids gave a greenish-blue color [19].

### The Meniran extract’s antibacterial activity against *S. pullorum* in vitro

#### Diffusion method

Meniran extract with 100% concentrate was obtained according to the dose by calculating the percentage (%) per volume of solution in grams put into a test tube containing a 10 mL 0.5% CMC Na solution (Merck). Meniran extract was used to make the treatments with 40%, 20%, 10%, and 5% concentrations. Prepared sterile NA medium and 3 mL *S. pullorum* suspension were poured into a Petri dish (Pyrex, Japan). Then, 20 mL NA medium was poured aseptically into a Petri dish while shaking regularly to form a homogeneous layer and allowed to half solidify. The six blank disks were put into the test medium and ignored for 15 min until the medium solidified. Then, Meniran extract solution tested from several concentrations (5%, 10%, and 20%) was placed into a blank disk installed on the test medium with as many as three drops. The control solution [2% tylosin antibiotic (Sigma, USA)] was pipetted and put into a blank disk attached to the test medium until the blank was full. One blank disk was used as a negative control with no solution added. It was incubated for 24 h at 37°C [20]. All tests were conducted in triplicates.

#### Diameter determination of the inhibitory zone

The diameter of the bacterial inhibition zone was a formed zone due to the activity from the active ingredient against the bacteria on agar media (Oxoid, UK). The bacterial inhibition zone was determined using a vernier caliper (Tricle Brand, China) in millimeters (mm). The diameter of the inhibition zone formed by Meniran extract would be compared with one formed by 2% tylosin antibiotics.

### Dilution method

#### Determination of minimum inhibitory concentration (MIC)

MIC was used to determine the minimum concentration of an antibacterial solution that could inhibit bacterial growth. MIC was performed by preparing 15 test tubes; tubes 1-3 were added with 3 mL 5% Meniran extract, tubes 4-6 with 3 mL 10% Meniran extract, tubes 7-9 with 3 mL 20% Meniran extract, tubes 10-12 with 3 mL 40% Meniran extract, and tubes 13-15 with 3 mL antibiotics as the positive control. Then, each concentration was combined with *S. pullorum* suspension (1×10^8^ CFU/mL) as much as 3 mL per concentration, then incubated at 37°C for 24 h. Consequently, the MIC results could be presented by observing the clarity or turbidity in the test tube of the Meniran extract before and after being incubated for 24 h at 37°C. MIC was defined by the result of the lowest concentration (mg/mL) of the Meniran extract resulting in clear broth media [21]. The MIC test results could be presented by observing the clarity or turbidity in the test tube of the Meniran extract before and after being incubated for 24 h at 37°C. MIC was confirmed by looking at the minimum bactericidal concentration (MBC) results.

#### Determination of MBC

MBC was aimed to decide the minimum concentration of an antibacterial solution that could kill bacteria in the media. MBC was conducted by implementing four plates of nutrient agar media. Each plate was divided into five parts; each part was a different concentration consisting of 40%, 20%, 10%, 5%, and positive control. The MIC test results were planted on nutrient agar media by scratching on each part of the media whose concentration had been determined and then incubated at 37°C for 24 h. Furthermore, the results can be observed by the existence or absence of the growth from *S. pullorum* colonies on the nutrient agar media.

### Meniran extract antibacterial activity against *S. pullorum* in vivo

This study had a completely randomized design. Twenty broilers were reared from the age of 1 day in brooder cages; fed, and watered using *ad libitum*. These chickens were then administered with ND vaccine through eye drops at the age of 4 days for the first line of defense. Then, at the age of 14 days, they were transferred to the battery cage for an adaptation period of 1 week before the given treatment. The broilers were divided into five equal groups consisting of four broilers per group. This division was based on the Federer formula [22]. Next, those chickens (except in the P0− group) were infected with *S. pullorum* with a concentration of 10^8^ cells/mL as much as 1 mL/head intramuscularly at the age of 14 days. Then, they were observed on which clinical symptoms were caused by *S. pullorum* infection at the age of 14-21 days. Moreover, their clinical symptoms began to appear on the 3^rd^ day after infection, which was at the age of 17 days, and all broilers were exposed to clinical symptoms on the 7^th^ day after infection, which was at the age of 21 days. Furthermore, Meniran extract was administered orally from day 21 to day 34 along with the calculation of their daily feed consumption and weight gain weekly. At the same time, their feed conversion was also measured after obtaining the data of feed consumption and weight gain. The treatment groups were as follows:

P0−: A group of four broilers that were neither administered with any Meniran extract nor *S. pullorum* but given distilled water as a substitute for the extract.

P0+: A group of four broilers that were unadministered with any Meniran extract but given with Aquades as a substitute for the extract and infected with *S. pullorum* intramuscularly with a concentration of 10^8^ cells/mL as much as 1 mL/head.

P1: A group of four broilers were provided with Meniran extract at a concentration of 5% as much as 1 mL/head orally and infected with *S. pullorum* intramuscularly with a concentration of 10^8^ cells/mL as much as 1 mL/head.

P2: A group of four broilers given 10% Meniran extract concentration as much as 1 mL/head orally and infected with *S. pullorum* intramuscularly with a concentration of 10^8^ cells/mL as much as 1 mL/head.

P3: A group of four broilers administered with a 20% Meniran extract concentration of as much as 1 mL/head orally and infected with *S. pullorum* intramuscularly with a concentration of 10^8^ cells/mL as much as 1 mL/head.

### Chicken performance calculation

Feed consumption was calculated daily by subtracting the given feed from the remaining or leftover feed. In contrast, the weight gain was calculated weekly, and the feed conversion was measured after getting the data on feed consumption and weight gain.

### Feed consumption

Calculation of feed consumption:

Feed consumption=Given feed (g)−Leftover feed (g)

### Weight gain

Calculation of weight gain:

Weight gain=Final body weight (g)−Initial body weight (g)

### Feed conversion

Feed conversion calculation:


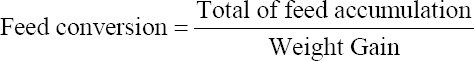


### Statistical analysis

The data from the phytochemical test were analyzed as descriptive. The data of the diffusion method were analyzed using one-way analysis of variance (ANOVA) and Duncan’s test. The data of the dilution method (MIC and MBC) were processed using probit analysis to determine LC_50_. The results of broilers’ performance were analyzed using ANOVA and Duncan’s test.

## Results and Discussion

### Meniran extract’s antibacterial activity against *S. pullorum* in vitro

**Figure-1:**
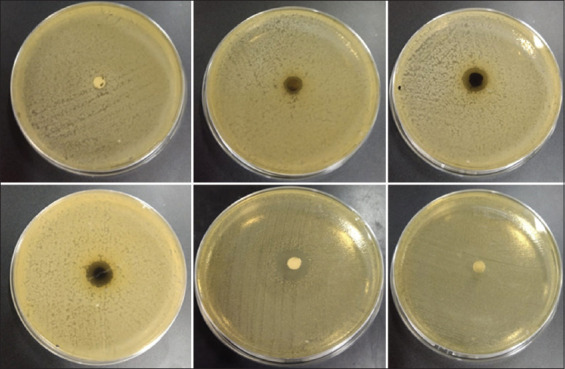
Diffusion results of Meniran extract. Description: The arrows point to the inhibition zone of each treatment.

The activity test results from Meniran extract against *S. pullorum* were positive. The results showed that 2% tylosin antibiotic formed the largest diameter of inhibitory zone from *S. pullorum* exposure, and the increased inhibitory zone correlated with the increasing of Meniran extract concentration. Thus, this was in accordance with the theory, which stated that the inhibition of microorganisms is directly proportional to the extract’s concentration [23]. The results of the data analysis are shown in Table-1. The positive results were indicated by a bacterial clear zone in brown. The results of the study are shown in Figure-1.

**Table 1:** Average and standard deviation of inhibitory zone diameter formed by the antibacterial Meniran extract against *Salmonella pullorum*.

Treatments	X±SD inhibitory zone (in millimeter)
P1: 40% Meniran extract	15.6^e^±0.57
P2: 20% Meniran extract	13.1^d^±0.81
P3: 10% Meniran extract	8.3^c^±0.48
P4: 5% Meniran extract	5.8^b^±0.17
P0+: 2% tylosin	19.3^f^±0.17
P0−: Blank disk	0^a^

^a, b, c, d, e, f^ Means in the same row with different superscripts differ significantly (p < 0.05), SD = Standard Deviation.

**Figure-2:**
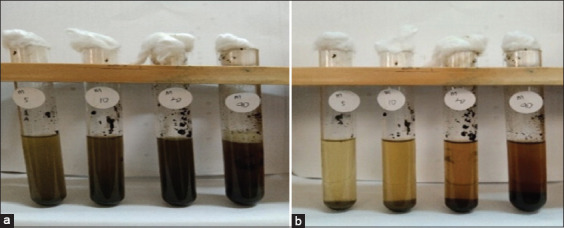
Minimum inhibitory concentration test of Meniran extract (a) before incubation and (b) after incubation.

**Figure-3:**
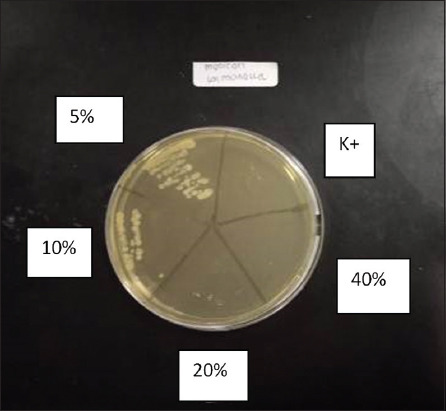
Results of dilution in Meniran extract.

The antibacterial activity of Meniran extract against *S. pullorum* could be displayed using the dilution method, which included MIC and MBC. The results of these examinations are shown in Table-2, Figures-2 and 3. The MIC test results could be presented by observing the clarity or turbidity in the test tube of the Meniran extract before and after being incubated for 24 h at 37°C. MIC results showed that 5% Meniran extract could already inhibit *S. pullorum* growth indicated by forming a clear area on the bacterial test media [24]. This meant that there was a bacterial growth inhibition.

**Table 2:** Minimum bactericidal concentration in Meniran and positive control antibiotics.

Cup number	Concentrate	Repetition			

		I	II	III	IV
1	5%	+	+	+	+
2	10%	+	+	+	+
3	20%	−	−	−	−
4	40%	−	−	−	−
5	K+	−	−	−	−

MBC results were obtained by observing the presence or absence of *S. pullorum* growth on the nutrient agar media. If it was indicated growth in the nutrient agar media, it demonstrated that Meniran extract could not kill the bacteria. If there was no bacterial growth, it indicated that Meniran extract could kill *S. pullorum*. The result of observation on the growth of *S. pullorum* displayed that Meniran extract at a dose of 20% indicated no *S. pullorum* growth.

*S. pullorum* growth still occurred at doses of 5% and 10% of Meniran extracts. Meanwhile, there was no *S. pullorum* growth at 20% and 40% Meniran extract doses. The antibacterial ability of Meniran extract is from the antibacterial compounds containing flavonoids, alkaloids, saponins, and tannins [11,12,25].

Moreover, the antibacterial working mechanism of Meniran extract began with the dismantling of the bacterial cell wall by flavonoids. Flavonoids that penetrated the cell wall caused damage to the permeability of bacterial cells as impaired permeability would cause the destroyed microsomes and lysosomes. Meanwhile, the mechanism of flavonoids as antibacterial was achieved by inhibiting nucleic acid synthesis, cytoplasmic membrane function, and bacterial energy metabolism [10,13,26].

Correspondingly, flavonoids are considered to possess antibacterial and antioxidant features that increase the work of the immune system because they can accelerate lymphoid and immune systems’ activation by producing leukocyte cells as antigen eaters. Besides, the working mechanism of flavonoids is to denature the proteins contained in the cell wall; therefore, they can transform the structure and mechanism of bacterial cell wall permeability [27].

Simultaneously, alkaloids can transform the genetic balance in the deoxyribonucleic acid chain, leading to damage of the bacterial DNA chain. Alkaloids are antibacterial substances that are effective in terminating and inhibiting Gram-negative and Gram-positive bacteria. Alkaloids act by destroying the constituent components of peptidoglycan in the bacterial cells; therefore, the bacterial wall layers would not be fully formed, resulting in bacterial cell death. Furthermore, alkaloids contained in Meniran extract serve as a performing mechanism to inhibit bacterial growth by forming complexes with bacterial proteins through hydrogen bonds, thus, inhibiting proteins and bacterial nucleic acids [28].

Furthermore, tannins contained in Meniran extract operate by impeding bacterial growth through the bacterial protoplasm coagulation. Tannins are useful for preventing microorganisms’ growth by precipitating the proteins from enzymes produced by microorganisms. Consequently, they become inactive and bacterial growth is hindered. Besides, tannins work with the mechanism related to their ability to inactivate the adhesion of bacterial cells and enzymes, along with interfering with the transport of protein in the cells’ inner layer. Moreover, tannins also have a target on the cell wall’s polypeptides. Therefore, cell wall formation is imperfect. This affects the bacterial cell to lyse due to the osmotic pressure; thus, the bacterial cell will die. Tannins are antibacterial by precipitating the proteins [10,11,28].

Other chemical substances useful in Meniran are saponins. As a mechanism, saponins harm the bacterial cell membranes by lowering the surface tension, resulting in increased cell membrane permeability and releasing important components, such as proteins, nucleic acids, and nucleotides, leading the bacteria to turn into lyses. Furthermore, saponins work by lysing the bacterial cell walls and disrupting the cell metabolism until death. In addition, saponins contained in Meniran extract will interact with the bacterial cell wall and affect the bacterial cell wall into lyses [12,13,28].

### Meniran extract’s antibacterial activity against *S. pullorum* in vivo

#### Feed consumption

**Figure-4:**
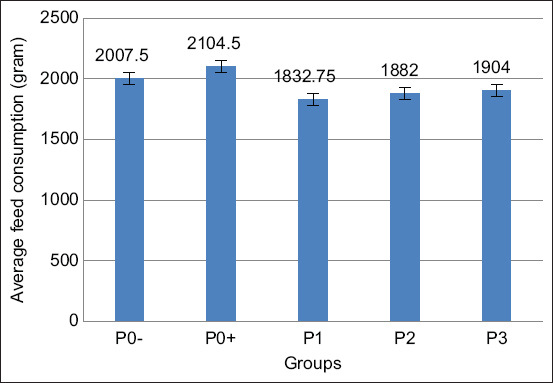
Average feed consumption in broiler chicken infected with *Salmonella pullorum* and administered with Meniran extract. P0-= Negative control treatment, P0+= Positive control treatment, P1= Treatment 1 (Group that was administrated with Meniran extract at 5% concentration), P2= Treatment 2 (Group that was administrated with Meniran extract at 10% concentration), P3= Treatment 3 (Group that was administrated with Meniran extract at 20% concentration). The administration of Meniran extract shows a decrease in feed consumption of broiler chickens infected with *Salmonella pullorum* than in control groups.

Based on the results of data analysis using ANOVA among treatment groups, that are shown in Table-3 and Figure-4, it was revealed that there was a significant difference (p<0.05) in the administration of Meniran extract on broiler feed consumption. Furthermore, the results of Duncan’s test also showed that the feed consumption of P0− and P0+ displayed no significant difference. Meanwhile, P0− and P0+ were significantly different from P1, and P0− exhibited a significant difference from P2. In addition, P0− was insignificantly different from P3 as well as P1, P2, and P3.

**Table 3:** Performance of the broilers.

Performance	Treatments Average±SD				

	P0−	P0+	P1	P2	P3
Feed consumption	2007.50^c^±125.51	2104.50^bc^±25.50	1832.75^a^±142.06	1882.00^ab^±55.42	1904.00^abc^±74.68
Weight gain	1261.25^c^±66.86	1145.50^abc^±129.36	1053.00^a^±103.66	1131.50^ab^±51.76	1179.50^b^±66.60
Feed conversion	1.59^a^±0.05	1.83^d^±0.01	1.74^c^±0.04	1.66^b^±0.02	1.61^a^±0.02

^a, b, c^ Means in the same row with different superscripts differ significantly (p<0.05), SD= Standard Deviation, P0-= Negative control treatment, P0+= Positive control treatment, P1= Treatment 1 (Group that was administrated with Meniran extract at 5% concentration), P2= Treatment 2 (Group that was administrated with Meniran extract at 10% concentration), P3= Treatment 3 (Group that was administrated with Meniran extract at 20% concentration).

Group P3 administered with the highest Meniran extract with a 20% concentration was insignificantly different from the P0− group. This proves that the level of feed consumption in P3 with the highest concentration administration could offset the level of feed consumption in P0−. Furthermore, Meniran extract effectively suppressed the growth of pathogenic bacteria and improved intestinal morphological characteristics because it has antibacterial and antioxidant effects, such as alkaloids, flavonoids, saponins, and tannins. Furthermore, Meniran extract can act as an immunostimulant; consequently, it can accelerate the healing process of diseases originating from bacteria or viruses, leading to the speeding up the process of being healthy and increasing the appetite. Moreover, the higher the dose given, the more compounds contained in the extract of Meniran are absorbed by the body of broilers.

Several factors can affect this study’s results, such as chicken’s type, strain, feed, and mild infections; therefore, the appetite of broilers infected with *S. pullorum* does not decrease. Correspondingly, the good level of feed consumption in broiler chicken is assumed to induce a condition in which the speed for repairing the damaged cells is faster than the decay of cells caused by *Salmonella*.

#### Weight gain

**Figure-5:**
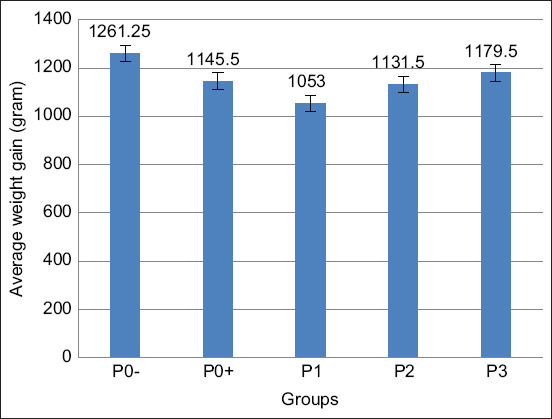
Average weight gain in broilers infected with *Salmonella pullorum* and administered with Meniran extract. P0-= Negative control treatment, P0+= Positive control treatment, P1= Treatment 1 (Group that was administrated with Meniran extract at 5% concentration), P2= Treatment 2 (Group that was administrated with Meniran extract at 10% concentration), P3= Treatment 3 (Group that was administrated with Meniran extract at 20% concentration). The administration of Meniran extract shows the gradual increase in weight gain of broiler chickens infected with *Salmonella pullorum*.

Based on the results from data analysis using ANOVA, that are shown in Table-3 and Figure-5, it exhibited a significant difference (p<0.05) between treatments administered with Meniran extract on the weight gain of broilers. Duncan’s follow-up test results showed that the weight gain of P0− was insignificantly different from that of P0+. However, P0− was significantly different from P1, P2, and P3; and P1 was significantly different from P3. Meanwhile, P2 was insignificantly different from P1 and P3; and P0+ was also insignificantly different from P1, P2, and P3.

Group P3 had a higher rate of body weight gain than Group P1 and P2. This is because of the direct proportionality of P3 feed consumption, having the highest feed consumption level compared to P1 and P2 treatments. Furthermore, feed consumption is directly proportional to body weight; if feed consumption increases, the achievement of body weight also increases. Thus, the changes in feed consumption caused the changes in chicken’s body weight. The higher consumption causes the higher protein that enters the animal’s body. As protein is the main constituent of body organs and soft tissues in various poultry animals, it is part of the enzymes in the body and antibodies needed in the meat’s growth, management, and production [29].

Meanwhile, Meniran extract is also effective as an antibacterial in impeding *Salmonella* bacterial growth. It can also improve the appearance of the intestinal villi, therefore, maximizing the digestibility of food substances from the consumed feed to meet the needs and consumption of broilers [30]. Thus, the weight gain of broilers will be maximal.

*S. pullorum* infection in broilers triggers the symptoms of white defecation and swelling in the leg joints. However, the damage induced by *S. pullorum* infection can remain tolerated by experimental animals; consequently, it does not provoke disturbances in broiler chicken’s body weight gain. Moreover, pullorum is an infectious disease in chickens, bringing very high mortality, especially in chicks aged 1-10 days. Meanwhile, in adult chickens, this disease generally rarely shows clear clinical signs and does not cause any death but serves as reservoirs; therefore, they can be transmitted to the healthy chickens vertically and horizontally [31].

#### Feed conversion

**Figure-6:**
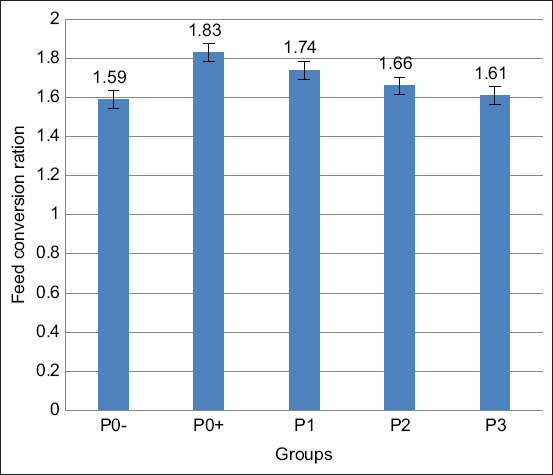
Average of feed conversion ratio in broilers infected with *Salmonella pullorum* and administered with Meniran extract. P0-= Negative control treatment, P0+= Positive control treatment, P1= Treatment 1 (Group that was administrated with Meniran extract at 5% concentration), P2= Treatment 2 (Group that was administrated with Meniran extract at 10% concentration), P3= Treatment 3 (Group that was administrated with Meniran extract at 20% concentration). The administration of Meniran extract shows a gradual decrease in feed conversion ratio of broiler chickens infected with *Salmonella pullorum* than the positive control group.

According to the data analysis, results using ANOVA are shown in Table-3 and Figure-6 explained a significant difference (p<0.05) between treatments related to the administered Meniran extract on the feed conversion of broilers. Duncan’s test results revealed that P0− and P3 feed conversion displayed no significant difference. P0− was significantly different from P0+, P1, and P2. Meanwhile, P3 was significantly different from P0+, P1, and P2.

The P3 treatment group with the highest dose of 20% Meniran extract was insignificantly different from the P0− treatment which was not given any Meniran extract and was uninfected with *S. pullorum*. This indicates that Meniran extract administration with a concentration of 20% has similar feed efficiency as the P0− treatment, which is the broiler without any Meniran extract administration and *S. pullorum* infection.

Furthermore, P3 with 20% Meniran extract concentration had a lower conversion rate than P1 with 5% Meniran extract and P2 with 10% Meniran extract. This means that P3 can suppress the feed conversion rate of broilers infected with *S. pullorum*. Accordingly, the higher concentration leads to a higher content of active substances; therefore, the antibacterial activity will be greater. The higher concentration of Meniran extract leads to the more contained secondary metabolites and the more bioactive compounds owned by Meniran, which have antibacterial activity, including flavonoids, terpenoids, alkaloids, saponins, and tannins. Furthermore, the herbal Meniran leaf extract has an antibacterial effect in restraining *Salmonella* bacterial growth. Consequently, broilers become healthier and their digestion gets better; thus, the consumed feed can be absorbed better, leading to increased growth while the feed conversion gets low [32,33].

Meanwhile, P0+ had the highest feed conversion rate because the amount of feed consumed was imbalanced with the body’s weight gain. The feed conversion value is expressed as a measure of feed efficiency, describing the livestock’s ability to convert feed into a certain amount of production in certain units, both for meat and egg production. Although in P0+, the feed consumption and body weight gain were undisturbed, it can be seen in the feed conversion that the ability of broilers to digest the feed to be converted into meat was poor because the feed conversion value remained high [34].

Similarly, the high and low feed conversion rates are due to the larger or lower difference in feed consumption ratio and body weight gain. The lower feed conversion rate contributes to a better condition because it shows that the use of feed becomes more efficient. The more efficient chicken is in converting its food into meat drives, the better conversion value will occur. If the conversion rate is high enough, the feed consumption will not be balanced with the resulting body weight gain [35].

Meniran extract is effective as an immunomod­ulator *in vivo* and as anti-inflammatory by preventing the nuclear factor-kB (NF-kB) production, and interleukin-8 induction factor as the main mediator for neutrophils. Besides, it can also reduce the tumor necrosis factor, an important factor in the acute inflammatory process, including neutrophil activation. In addition, the mechanism of the anti-inflammatory effect from Meniran extract is demonstrated through the ability to decrease nitric oxide synthesis induced with endotoxin and cyclooxygenase along with preventing NF-kB production [36,37].

## Conclusion

Meniran extract has shown to contain alkaloids, tannins, flavonoids, saponins, steroids, and triterpenoids. It also has the ability as an antibacterial against *S. pullorum*, with the largest inhibitory zone average at a dose of 40% is 15.6 mm, 13.15 mm for 20%, 8.38 mm for 10%, and 5.8 mm for 5%. Furthermore, Meniran extract possesses an antibacterial feature against *S. pullorum* with an MBC of 20% concentration. Finally, administering Meniran extract to broilers infected with *S. pullorum* can increase the consumption value, weight gain, and reduce the broilers’ value of feed conversion ratio. The best results are in the treatment using Meniran extract with 20% concentration. This study shows that Meniran can be used to antagonize *S. pullorum* on broilers and improve the performance of broilers infected with the bacteria. The data of this study can be used as a reference for future studies on the use of Meniran as an antibacterial against *S. pullorum* on other poultry birds.

## Authors’ Contributions

SH: Concept and designed the study, analysis and interpretation of data, and writing and critical review of the manuscript. EKS: Concept and designed the study, data acquisition, and writing and critical review of the manuscript. KR: Concept and designed the study and critical review of the manuscript. SS: Concept and designed the study, acquisition, analysis and interpretation of data, and critical review of the manuscript. GGAT, MAH, and TPW: Data acquisition and critical review of the manuscript. All authors read and approved the final manuscript.

## References

[1] Wang Y, Huang C, Tang J, Liu G, Hu M, Kang X, Zhang J, Zhang Y, Pan Z, Jiao X, Geng S (2021). *Salmonella pullorum* spiC mutant is a desirable LASV candidate with proper virulence, high immune protection and easy-to-use oral administration. Vaccine.

[2] Cheng Y, Sihua Z, Lu Q, Zhang W, Wen G, Luo Q, Shao H, Zhang T (2020). Evaluation of young chickens challenged with aerosolized *Salmonella pullorum*. Avian Pathol.

[3] Guo R, Geng S, Jiao H, Pan Z, Chen X, Jiao X (2016). Evaluation of protective efficacy of a novel inactivated *Salmonella pullorum* ghost vaccine against virulent challenge in chickens. Vet. Immunol. Immunopathol.

[4] Barrow P.A, Freitas Neto O.C (2011). Pullorum disease and fowl typhoid--new thoughts on old diseases:A review. Avian Pathol.

[5] Zhang D, Zhuang L, Wang C, Zhang P, Zhang T, Shao H, Han X, Gong J (2018). Virulence gene distribution of *Salmonella pullorum* isolates recovered from chickens in China (1953-2015). Avian Dis.

[6] Hidanah S, Sabdoningrum E.K, Wahjuni R.S, Chusniati S (2018). Effects of meniran (*Phyllanthus niruri* L.) administration on leukocyte profile of broiler chickens infected with *Mycoplasma gallisepticum*. Vet. World.

[7] Lee N.Y, Khoo W.K, Adnan M.A, Mahalingam T.P, Fernandez A.R, Jeevaratnam K (2016). The pharmacological potential of *Phyllanthus niruri*. J. Pharm. Pharmacol.

[8] Zheng Z.Z, Chen L.H, Liu S.S, Deng Y, Zheng G.H, Gu Y, Ming Y.L (2016). Bioguided fraction and isolation of the antitumor components from *Phyllanthus niruri* L. Biomed. Res. Int.

[9] Geethangili M, Ding S.T (2018). A review of the phytochemistry and pharmacology of *Phyllanthus urinaria* L. Front. Pharmacol.

[10] Beidokhti M.N, Andersen M.V, Eid H.M, Villavicencio M.L.S, Staerk D, Haddad P.S, Jäger A.K (2017). Investigation of antidiabetic potential of *Phyllanthus niruri* L. using assays for ?-glucosidase, muscle glucose transport, liver glucose production, and adipogenesis. Biochem. Biophys. Res. Commun.

[11] Kaur N, Kaur B, Sirhindi G (2017). Phytochemistry and pharmacology of *Phyllanthus niruri* L.:A review. Phytother. Res.

[12] Ezzat M.I, Okba M.M, Ahmed S.H, El-Banna H.A, Prince A, Mohamed S.O, Ezzat S.M (2020). In-depth hepatoprotective mechanistic study of *Phyllanthus niruri*:*In vitro* and *in vivo* studies and its chemical characterization. PLoS One.

[13] Londhe J.S, Devasagayam T.P, Foo L.Y, Shastry P, Ghaskadbi S.S (2012). Geraniin and amariin, ellagitannins from *Phyllanthus amarus*, protect liver cells against ethanol-induced cytotoxicity. Fitoterapia.

[14] Hidanah S, Sabdoningrum E. K, Wahjuni R. S, Chusniati S (2018). Effects of meniran (*Phyllanthus niruri* L.) administration on leukocyte profile of broiler chickens infected with *Mycoplasma gallisepticum*. Vet. World.

[15] Hidanah S, Sabdoningrum E. K, Wahyuni R. S, Dewi A. R (2018). Effectiveness of meniran (Phyllanthus niruri Linn) as antibacterial for resistance antibiotics prevention of enterotoxin Escherichia Coli. Indones. J. Trop. Infect. Dis.

[16] Sabdoningrum E.K, Hidanah S, Ansori A.N.M, Fadholly A (2020). Immunomodulatory and antioxidant activities of *Phyllanthus niruri* L. extract against the laying hens infected by *Escherichia coli*. Res. J. Pharm. Technol.

[17] Kurnijasanti R, Putri A.A (2016). The effects of banana stem (*Musa Paradisiaca* var. Sapientum) extract on histopathologic gastric of rats induced by indomethacin. Folia Med. Indones.

[18] Muchtaromah B, Wahyudi D, Ahmad M, Ansori A.N.M, Annisa R, Hanifah L (2021). Chitosan-tripolyphosphate nanoparticles of mango ginger (*Curcuma mangga*) extract:Phytochemical screening, formulation, characterization, and antioxidant activity. Phcog. J.

[19] Sabdoningrum E.K, Hidanah S, Chusniati S (2021). Characterization and phytochemical screening of meniran (*Phyllanthus niruri* Linn.) extract's nanoparticles used Ball Mill method. Phcog. J.

[20] Hidanah S, Sabdoningrum E.K, Al Arif M.A, Ansori A.N.M, Hasanah T.P, Widaya L.V.A (2020). Sambiloto (*Andrographis paniculata*) extract improves the performance of animal model infected with *Escherichia coli*. Indian J. Forensic. Med. Toxicol.

[21] Faden A.A (2018). Evaluation of antibacterial activities of aqueous and methanolic extracts of areca catechu against some opportunistic oral bacteria. Biosci. Biotechnol. Res. Asia.

[22] Husen S.A, Winarni D, Ansori A.N.M, Susilo R.J.K, Hayaza S, Darmanto W (2020). Antioxidant potency of okra (*Abelmoschus esculentus* Moench) pods extract to ameliorate kidney structure and function in diabetic mice. Econ. Environ. Cons.

[23] Kumar M, Saurabh V, Tomar M, Hasan M, Changan S, Sasi M, Maheshwari C, Prajapati U, Singh S, Prajapat R.K, Dhumal S, Punia S, Amarowicz R, Mekhemar M (2021). Mango (*Mangifera indica* L.) leaves:Nutritional composition, phytochemical profile, and health-promoting bioactivities. Antioxidants.

[24] Samudra D, Santoso S, Asmoro A.A, Khotimah H, Ansori A.N.M, Sabdoningrum E.K (2021). The effect of *Escherichia coli* on pro-inflammatory mediators level and kidney and liver function of sepsis in *Rattus novergicus*. Indian J. Forensic. Med. Toxicol.

[25] Zhou J, Bi S, Chen H, Chen T, Yang R, Li M, Fu Y, Jia A.Q (2017). Anti-biofilm and antivirulence activities of metabolites from *Plectosphaerella cucumerina* against *Pseudomonas aeruginosa*. Front. Microbiol.

[26] Sun F, Li X, Wang Y, Wang F, Ge H, Pan Z, Xu Y, Wang Y, Jiao X, Chen X (2021). Epidemic patterns of antimicrobial resistance of *Salmonella enterica* serovar Gallinarum biovar Pullorum isolates in China during the past half-century. Poult. Sci.

[27] Nhung N.T, Chansiripornchai N, Carrique-Mas J.J (2017). Antimicrobial resistance in bacterial poultry pathogens:A review. Front. Vet. Sci.

[28] Legba B, Dougnon V, Chabi Y, Gbaguidi C, Aniambossou A, Deguenon E, Dougnon J, Kpodekon M, Baba-Moussa L (2020). Evaluation of *in-vivo* anti-Salmonella activity of *Uvaria chamae*, Lantana camara and *Phyllantus amarus* used in Benin, West Africa. BMC Vet. Res.

[29] Raji M.A, Kazeem H.M, Magyigbe K.A, Ahmed A.O, Lawal D.N, Raufu I.A (2021). *Salmonella* serovars, antibiotic resistance, and virulence factors isolated from intestinal content of slaughtered chickens and ready-to-eat chicken gizzards in the Ilorin metropolis, Kwara State, Nigeria. Int. J. Food Sci.

[30] Deng Z, Han D, Wang Y, Wang Q, Yan X, Wang S, Liu X, Song W, Ma Y (2021). *Lactobacillus casei* protects intestinal mucosa from damage in chicks caused by *Salmonella pullorum* via regulating immunity and the Wnt signaling pathway and maintaining the abundance of gut microbiota. Poult. Sci.

[31] Foley S.L, Johnson T.J, Ricke S.C, Nayak R, Danzeisen J (2013). *Salmonella* pathogenicity and host adaptation in chicken-associated serovars. Microbiol. Mol. Biol. Rev.

[32] Pulido-Landínez M, Sánchez-Ingunza R, Guard J, do Nascimento V.P (2014). Presence of *Salmonella enteritidis* and *Salmonella gallinarum* in commercial laying hens diagnosed with fowl typhoid disease in Colombia. Avian Dis.

[33] Kang M.S, Kim A, Jung B.Y, Her M, Jeong W, Cho Y.M, Oh J.Y, Lee Y.J, Kwon J.H, Kwon Y.K (2010). Characterization of antimicrobial resistance of recent *Salmonella enterica* serovar Gallinarum isolates from chickens in South Korea. Avian Pathol.

[34] Wang Y, Li J, Xie Y, Zhang H, Jin J, Xiong L, Liu H (2021). Effects of a probiotic-fermented herbal blend on the growth performance, intestinal flora and immune function of chicks infected with *Salmonella pullorum*. Poult. Sci.

[35] Bae W.Y, Kim H.Y, Yu H.S, Chang K.H, Hong Y.H, Lee N.K, Paik H.D (2021). Antimicrobial effects of three herbs (*Brassica juncea*, *Forsythia suspensa*, and *Inula britannica*) on membrane permeability and apoptosis in *Salmonella*. J. Appl. Microbiol.

[36] Tie K, Yuan Y, Yan S, Yu X, Zhang Q, Xu H, Zhang Y, Gu J, Sun C, Lei L, Han W, Feng X (2018). Isolation and identification of *Salmonella pullorum* bacteriophage YSP2 and its use as a therapy for chicken diarrhea. Virus Genes.

[37] Yin C, Liu Z, Xian H, Jiao Y, Yuan Y, Li Y, Li Q, Jiao X (2020). AvrA exerts inhibition of NF-?B pathway in its naïve *Salmonella* serotype through suppression of p-JNK and beclin-1 molecules. Int. J. Mol. Sci.

